# Assessment of alcohol utilization during pregnancy and its associated factors among reproductive women in Mecha Woreda of North Western Ethiopia

**DOI:** 10.1186/s12905-022-01776-0

**Published:** 2022-05-25

**Authors:** Getaneh Bizuayehu Demeke, Eyayu Kasseye Bayu

**Affiliations:** 1 Department of Population Studies, College of Social Sciences and Humanities, University of Gondar, Gondar, Ethiopia; 2 Department of Gender and Development Studies, College of Social Sciences and Humanities, University of Gondar, Gondar, Ethiopia

**Keywords:** Alcohol use, Ethiopia, Mecha Woreda, Pregnancy, Reproductive women

## Abstract

**Background:**

Pregnancy is a time when women are making many changes, including the patterns of alcohol consumption. Alcohol consumption during pregnancy encourages the risks of mothers and unborn child. Alcohol use during pregnancy can result in prematurity, brain damage, growth restriction, developmental delay and social, emotional and behavioral deficits, particularly in developing countries.

**Methods:**

A community based cross-sectional study was employed. Structured questionnaires were used to assess the prevalence of alcohol utilization, and socio-demographic as well as economic characteristics of women who have pregnancy experience. Both bivariate and multivariate logistic regression models were employed. Descriptive and inferential statistical analyses were used.

**Results:**

The study results showed that the prevalence of alcohol use and response rate was high. Factors like age group of women from 35–49 years (AOR = 0.221; 95%CI = 0.057–0.856), illiterate women(AOR = 2.697;95% CI = 1.207–6.026), currently pregnant (AOR = 0.139;95%CI = 0.057 0.343), women currently use alcohol (AOR = 0.021; 95% CI = 0.009 0.049), alcohol use pre-pregnancy (AOR = 0.016; 95% CI = 0.006–0.042), women drinking alcohol with husband during pregnancy (AOR = 0.228; 95% CI = 0.085–0.614), the risk of alcohol consumption during pregnancy is low(AOR = 0.262;95%CI = 0.074–0.925), risk alcohol consumption during pregnancy is medium (AOR = 0.296;95% CI = 0.103–0.849),utilization of alcohol during pregnancy is valuable (AOR = 0.104; 95%CI = .0.013–0.833) were statistically associated with alcohol use during pregnancy.

**Conclusion:**

The result inferred that there is a high level of alcohol use throughout pregnancy. Due to the differences in the culture and communal means of drinking alcohol, the frequency of alcohol consumption during pregnancy varies among different regional studies and countries.

## Background

Pregnancy can be the cause of various changes in women, including alterations in their alcohol intake behaviors. During pregnancy, all types of alcoholic beverages can be detrimental to the fetus, and the risk is proportional to the amount of alcohol taken [1]. The use of alcohol is responsible for 3.3 million fatalities worldwide each year [2].

Miscarriage, early birth, stillbirth, and high blood pressure are all linked to excessive alcohol consumption. They may also vomit more frequently and be dehydrated [3]. Maternal mortality continues to be a major public health issue. The majorities of these deaths are avoidable and linked to modifiable risk factors. Alcohol consumption during pregnancy is one of the few avoidable and controllable risk characteristics for poor pregnancy and delivery outcomes [4]. According to the study, alcohol intake during pregnancy is higher among African women than in other parts of the world [5]. In both developed and developing countries, a pregnant woman with low socio- economic level was found to be more vulnerable to alcohol and other drugs use [6].

The study carried out in US demonstrated that, the level of any substance use during pregnancy was 25.8% just as cigarette and alcohol usage were 18.9 and 10%, respectively. The employed and married women were essentially more averse to devour any substance during pregnancy, looked at the age, nationality and income [7]. The review led in Ghana on pregnant women shown that, 22% of the respondents were uninformed about the unfavorable wellbeing impacts of alcohol utilization during pregnancy, and 45% of them had not gotten training on the adverse consequences of alcohol on the mother or the baby [8].

The home-made and factory-made alcoholic drinks are used in Ethiopia. The estimated alcohol content for extraordinary traditional alcoholic drinks were (2–4%) for “Tella,” (7–11%) for “Tej” and (45%) for “Areki” [9]. Normally, alcohol intake all through being pregnant is a common danger element for the health of new infant and mother. As findings of mounting evidences indicated, previous studies focus on prevalence of alcohol use and studied only on pregnant women in either of rural or urban areas on institutional based survey. Here, this study is intended to address all gaps which did not address in terms of study subjects, area coverage, objectives and study approach (both pregnant women and non-pregnant women, rural and urban areas, prevalence and its associated factors using community based household study were included. Consequently, this has aimed to assess the prevalence of alcohol use during pregnancy and its associated factors among reproductive women (15–49) in Mecha Woreda of North West Ethiopia.

The severity of the problem and state of knowledge: The prevalence of alcohol use is severe in rural areas where the majority of women are dying, and pregnant women face abortion and complicated reproductive health problems. Persistent cultural norms, societal attitudes, and related factors lead to a non-stop increment of maternal mortality and child morbidity due to alcohol use and non-availability of research dissemination and interventions. So many additional variables are included in this study based on the context of both rural and urban areas in the study area. However, the results of this study would play a paramount role in immediate and long term interventions for health care institutions and organizations working on women’s health issues.

## Methods

### Setting and design of the study

This study was conducted from November 2020 to August 2021 using a community-based cross- sectional study design. The investigation was conducted in the Mecha woreda in northwestern Ethiopia. It was 529 km away from Addis Ababa and 34.2 km away from Bahir Dar. This woreda is found in the north of Bahir Dar Zuria woreda, in the South Mecha woreda in the south, the South Achefer woreda in the west, and in the Yilmana Densa woreda in the east. Mecha woreda included 33 rural and 3 urban kebeles. The vast bulk of the population lives in rural areas (80.6%).

The study's inclusion criteria were reproductive women [aged 15 to 49]. However, the non- pregnant women were included to know the extent of alcohol utilization in pregnancy experience, and they were also part of the reproductive age to generalize reproductive women’s alcohol usage and their determinants. The other basic concern is the inability of enough pregnant women to sample a unit while conducting this study on the site, and it is difficult to find out the alcohol experience of previous pregnant women. The data were collected via train enumerators, told the aims of the study and research questions carefully, and performed appropriate data collection tools to minimize the recall bias. The ethics committee of the college of social sciences and humanities research and publication coordination office at the University of Gondar provided the written consent. Study participants gave verbal informed consent after we explained the study's voluntary nature, their right to withdraw from it at any time without penalty, the participants' right to freedom of choice and expression, and the study's anonymity and confidentiality.

### Context and sampling procedures

To identify the study subjects in advance of sampling, the researchers used multistage sampling approaches. In addition, basic random sampling was utilized to define zones within a region and Woreda within the zones. As a result, Mecha Woreda was chosen at random based on this procedure. In addition, after selecting the eligible groups in Woreda reproductive women, a systematic sampling technique was used to identify the required sample. They were chosen through a systematic random selection from the existing health center and hospital registrar sampling frames. Following the selection of study participants, reproductive women were chosen at random from selected Woreda Kebeles every n^th^ interval. Then, as a starting point, we selected reproductive women as a reference. Finally, eligible reproductive women were invited to participate in this study, which was followed by the same protocols. The sample size was then calculated using a single population proportion formula and the following assumptions:

Take a 95% level of confidence with a 0.05 value (which generates Z/2 = 1.96 on the standard normal distribution curve), a 5% margin of error (d = 0.05), and the proportion of pregnant women who drink alcohol. A 34% usage rate was obtained from a prior study among reproductive women in Bahir Dar [10], with a 10% contingency. The formula is as follows: (Z/2)2 p (1-p)/ (d2) [11]. Where ‘n’ denotes the needed sample size and p denotes the prevalence of alcohol consumption during pregnancy. Likewise, 34% = 0.34 d2 = margin of error = 0.05, Z/2 = confidence level equivalent to 95 percent CI = 1.96 (= 0.05) and contingencies 10% 0.5 *n* = (1.96)2 0.34(1–0.34)/ (0.05)2) = 345 + (345*0.1) = 380. As a result, it is determined based on the information provided above.

### Data collection tools

The study focuses on issues such as demographics, socioeconomic status, and alcohol use during pregnancy among reproductive women. Age, married status, educational status, place of residence, religious affiliation, employment position, and average monthly income of households are all included in the socio-economic and demographic information. Alcohol utilization was also mentioned in the questionnaire. These include having ever used it, currently using it, being pregnant, the frequency with which you have used it, drinking with your husband while pregnant, having used alcohol before pregnancy, and the sorts of alcoholic drinks you have consumed. All of this was measured using dichotomous "yes/no" questions, with the exception of the frequency of use. However, the frequency of alcohol consumption during pregnancy was assessed using the following criteria: every day, 1–2 days a week, 2–3 days a week, 3–4 days a week, and once a week. The data were collected using a structured questionnaire, which was designed based on available information obtained from various literature sources. For the data gathering process, the questionnaire was first written in English and then translated into Amharic. The data collection technique involved six trained data collectors and two supervisors. Supervisors accompanied the data collectors and made any necessary adjustments on the spot.

### Data management, analysis and quality assurance

The descriptive statistics were employed, including frequency (*N*), percentage (%), mean, and standard deviation (SD). Individual level characteristics that were significantly linked with various forms of alcohol consumption during pregnancy were identified using a bivariate test. Then, multivariate logistic regression analysis with a bivariate p-value of 0.25 was used. To find the predictors of alcohol use during pregnancy, the researchers used a multivariate logistic regression analysis. Multivariate logistic regression findings are provided as AOR with a 95 percent confidence range (95 percent CI). The significance level was set at *p* < 0.05. SPSS 24 was used to analyze the quantitative data, which was entered, cleaned, and analyzed.

All ethical issues were taken into account in all procedures of this research. Here, the ethics committee of the College of Social Science and Humanities Research and Publication Coordination Office gave their approval to the project. All study participants gave their informed consent, and there were no individuals under the age of 15 in this study. In addition to this purpose and the importance of the study was explained and informed consent was obtained from the parents/guardians of the minors and from each illiterate participants (below the age of 18). The participants were included based on voluntarism and agreement. Participants in the study ranged in age from 15 to 49 years old, since they are briefly explained and understand the questions in well manner. All procedures were carried out in line with the Helsinki Declaration.

## Results

This chapter deals with the demographic characteristics, the prevalence of alcohol utilization, and variables that determine alcohol utilization during pregnancy in the Mecha Woreda Amhara Region. It was presented in three parts. Furthermore, it consists of demographic characteristics, the prevalence of alcohol utilization, and its determinant factors. It was analyzed using descriptive statistics such as frequency, percentage and inferential statistics such as binary logistic regression and multivariate analysis.

### Socio-demographic and economic characteristics of respondents in the study area

A total of three hundred eighty questionnaires were distributed, and the majority of respondents were appropriately filled. The mean age of the respondents was 35.22 (± 7.359), with an age ranging from 18 to 49 years. As it can be seen from Table 1, the age of the respondents was classified into three age categories: 15–24, 25–34, and 35–49. From these age categories, the majority the respondents were found between the age groups of 35 and 49, followed by those aged 25–34. As far as the marital status of the respondents was concerned, a large proportion of the respondents were married women. It can be understood that the majority of the study participants from the marital status category were married. In terms of residence, half of the respondents were rural, while the other half were urban.

**Table 1:** Socio-demographic and economic characteristics of study participants, in Mecha Woreda, Northwest, Ethiopia, 2021. *Source*: Obtained from survey data, 2021

Variables	Options	Frequency (*N*)	Percentage (%)
Age	15–24	24	6.4
	25–34	150	40.1
	35–49	200	53.5
Marital statues	Single	4	1.1
	Married	346	92.5
	Divorced	16	4.3
	Widowed	8	2.1
Place of residence	Rural	187	50
	Urban	187	50
Religious affiliation	Orthodox	353	94.4
	Muslim	18	4.8
	Protestant	3	0.8
Educational status	Illiterate	170	45.5
	Literate	204	54.5
Occupational status	Government employee	49	13.1
	Housewife	248	66.3
	Own business	72	19.3
	Daily laborer	5	1.3
Average monthly income of households in USD	9.74–48.69 USD	259	69.2
	48.71–97.37USD	108	28.9
	97.39–146.64USD	7	1.9

Based on the religious affiliations of the study participants, the majority of respondents were Orthodox, followed by Muslims, and Protestants, in descending order, respectively. Concerning the educational level, a few study participants were illiterate in their educational status, while the majority of respondents were literate. The occupational status of the respondents also determines the amount of alcohol utilized during pregnancy. Of the study participants, the majority of study participants were housewives, followed by those engaged in their own businesses, while a small number of respondents were working as daily laborers. Finally, according to the study findings, the study respondents have an average monthly income of 9.74–48.69 USD, 48.71–97.37 USD, and 97.39–146.64 USD, in descending order, respectively (Table 1).

### The prevalence of alcohol utilization during pregnancy in the study area

As shown in Table 2, the prevalence of alcohol use during pregnancy was high. According to these findings, a majority of respondents have used alcohol during pregnancy; which might have affected women’s as well as children’s health. The result showed that a small number of women were currently pregnant, while the majority were not currently pregnant. With regard to the current use of alcohol, a large number of the respondents currently use alcohol, while a small number of them do not. In terms of how frequently they drink alcohol now, the majority of study participants drink 1–2 days per week, with only a small percentage drinking every day.

**Table 2:** The prevalence of alcohol utilization during pregnancy, in Mecha Woreda, Northwest, Ethiopia, 2021. *Source*: Obtained from survey data, 2021

Variables	Options	Frequency (*N*)	Percentage (%)
Ever used alcohol during pregnancy	Yes	245	65.5
	No	129	34.5
Pregnant currently	Yes	90	24.1
	No	284	75.9
Currently, use alcohol	Yes	287	76.7
	No	87	23.3
How often you drink alcohol currently	Everyday	9	3.1
	1–2 days a week	108	37.6
	2–3 days a week	92	32.1
	3–4 days a week	40	13.9
	Once a week	38	13.2
Drinking Tella	Yes	259	90.2
	No	28	9.8
Drinking Areki	Yes	107	37.3
	No	180	62.7
Drinking Tej	Yes	6	2.1
	No	281	97.9
Drinking wine	Yes	17	5.9
	No	270	94.1
Drinking beer	Yes	13	4.5
	No	274	95.5
Used alcohol pre-pregnancy	Yes	257	68.7
	No	117	31.3
Drinking alcohol with husband during pregnancy	Yes	154	41.2
	No	220	58.8
Motivated your friends to drink alcohol during pregnancy	Yes	51	13.6
	No	323	86.4
Motivated your relatives to drink alcohol during pregnancy	Yes	37	10
	No	337	90
From experiences, how do you see the risk of alcohol consumption during pregnancy	Low	88	23.5
	Medium	214	57.2
	High	72	19.3
Miscarriage, high blood pressure, premature birth and others occur within your family	Yes	113	30.2
	No	261	69.8
Utilize of alcohol during pregnancy is valuable	Agree	28	7.5
	Strongly agree	7	1.9
	Dis agree	214	57.2
	Strongly disagree	29	7.8
	Neutral	96	25.7

Among the alcohol utilization practices, the majority of the respondents drink Tella (local beer), while a small number of them do not drink it. The result shows a small number of the study participants drink Areki (local beer), while a great number of them do not drink it. Tej was consumed by some respondents, while many of the study participants did not drink it. Furthermore, some of the study respondents drank wine, while the majority of respondents did not drink. It can be understood that the majority of the respondents do not drink wine. Last but not least, a small number of the respondents drink beer, while the majority of them do not drink beer.

The study findings revealed that in times of pre-pregnancy, a significant number of the respondents consumed alcohol in pre-pregnancy, while a small number of them did not. The study indicated that, in the cases of drinking alcohol with a husband during pregnancy, a small number of the respondents drank alcohol before the pregnancy, while the majority of the respondents did not drink it. As far as friend’s motivation to drink alcohol during pregnancy is concerned, a slight number of respondents have been motivated by friends to drink alcohol during pregnancy, while the majority of the respondents were not motivated by friends to drink. Another result also showed that some of the respondents had been encouraged to drink alcohol during pregnancy by relatives, while the majority of them had not been motivated by relatives to drink alcohol. This implies that the majority of the respondents drink alcohol in times of pregnancy because of the very nature and personal behaviors of those who drink alcohol, rather than of their husbands, friends, and relatives motivations (Table 2).

### Determinants of alcohol utilization during pregnancy in the study area

The Binary Logistic Regression Model (BLRM) was employed to establish a relationship between the utilization of alcohol during pregnancy and a set of explanatory variables. As Hulsizer and Woolf noted, binary logistic regression has become the preferred tool for predicting dichotomous outcomes in the social sciences because it is more flexible than any other model [12]. In the study area, the binary logistic regression model was employed to establish the relationship between dependent (utilization of alcohol during pregnancy) and independent variables (socio-demographic and economic factors) affecting utilization of alcohol during pregnancy.

The variables associated with significance in bivariate logistic analysis were exported to multivariate logistic regression analysis. Those variables significantly associated in the bi-variate analysis were women’s age category from 25–34 and 35–49 years old, rural residence, illiterate women, average monthly income of households from 48.71–97.37USD, pregnant currently (yes), currently use alcohol (yes), use alcohol pre-pregnancy (yes), drinking alcohol with husband during pregnancy (yes), miscarriage, high blood pressure, premature birth, and others occurring within your family (yes), attitude towards the risk of alcohol consumption during pregnancy (low and medium) and utilization of alcohol during pregnancy is valuable, agreed, disagreed, and strongly disagreed. Then, these variables were entered into a multivariate logistic regression analysis to adjust for confounding factors. Then, the following variables were significantly associated with the prevalence of alcohol utilization during pregnancy among reproductive women at a *p*-value < 0.05.Like women’s age category of 35–49 years old, educational status (illiterate), pregnant currently (yes), currently using alcohol (yes), using alcohol pre-pregnancy (yes), drinking alcohol with husband during pregnancy (yes), attitude towards the risk of alcohol consumption during pregnancy (low and medium), using alcohol during pregnancy is valuable (agree) were determinant factors influencing the dependent variable. Regarding the model goodness of fitness, the Hosmer and Lemeshow test, with a chi-square value of 11.601, df = 8, and a value of 0.170, which is significant at *p* > 0.05, indicated that it has goodness of-fit (Table 3).

**Table 3:** Bivariate and multivariate logistic regression analysis for factors associated with the prevalence of alcohol use during pregnancy among reproductive women, in Mecha Woreda, North West, Ethiopia, 2021. *Source*: Obtained from survey data, 2021

Variables	Ever used alcohol during pregnancy		Odds ratio(OR)		
	Yes	No	B	COR (95%CI)	AOR (95%CI)
*Age*					
15–24	8	16		1	1
25–34	95	55	− 1.240(− 0.987)	0.289 (0.116–0.720) 0.008	0.373(0.096–1.439) 0.152
35–49	142	58	− 1.589(− 1.508)	0.204(0.083–0.503)0.001	0.221(0.057–0.856) 0.029
*Place of residence*					
Rural	138	49	− 0.745(− 0.772)	0.475(0.307–0.735) 0.001	0.462(0.205–1.040) 0.062
Urban	107	80		1	1
*Educational status*					
Illiterate	131	39	0.975(0.992)	2.652(1.688–4.167)0.000	2.697(1.207–6.026)0.016
Literate	114	90		1	1
*Average monthly income of households in USD*					
9.74–48.69 USD	186	73		1	1
48.71–97.37USD	56	52	0.861(0.069)	2.366(1.487–3.765)0.000	1.072(0.520–2.209) 0.851
97.39–146.64USD	3	4	1.223(− 0.646)	3.397(0.742–15.552)0.115	0.524(0.028–9.729) 0.665
*Pregnant currently*					
Yes	71	19	− 0.860(− 1.971)	0.423(0.242–0.741)0.003	0.139(0.057–0.343)0.000
No	174	110		1	1
*Currently, use alcohol*					
Yes	232	55	− 3.179(− 3.843)	0.042(0.022–0.080)0. 000	0.021(0.009–0.049) 0.000
No	13	74		1	1
Use alcohol pre-pregnancy					
Yes	234	23	− 4.585(− 4.125)	0.010(0.005–0.022) 0.000	0.016(0.006–0.042)0.000
No	11	106		1	1
*Drinking alcohol with husband during pregnancy*					
Yes	147	7	− 3.264(-1.478)	0.038(0.017–0.085)0.000	0.228(0.085–0.614) 0.003
No	98	122		1	1
*Miscarriage, high blood pressure, premature birth and others occur within your family*					
Yes	94	19	− 1.282(0.356)	0.277(0.160–0.481) 0.000	0.700(0.279–1.758) 0.448
No	151	110		1	1
*From experiences, how do you see the risk of alcohol consumption during pregnancy*					
Low	60	28	− 1.099(− 1.341)	0.333 (0.174–0.638)0.001	0.262(0.074–0.925) 0.037
Medium	155	59	1.302( − 1.219)	0.272(0.156–0.474)0.001	0.296(0.103–0.849) 0.024
High	30	42		1	1
*Utilize of alcohol during pregnancy is valuable*					
Agree	26	2	− 1.521(− 2.263)	0.218(0.048–0.988)0.048	0.104(0.013–0.833) 0.033
Strongly agree	6	1	− 0.748(− 1.236)	0.473(0.054–4.127)0.498	0.291(0.012–7.127) 0.449
Disagree	132	82	0.568(− 0.103)	1.764(1.036–3.005)0.037	0.902(0.349–2.332) 0.832
Strongly disagree	10	19	− 1.686(1.269)	5.396(2.213–13.155) 0.000	3.557(0.844–14.987) 0.084
Neutral	71	25		1	1

## Discussions

The purpose of this study is to determine the prevalence of alcohol utilization during pregnancy and its associated factors among reproductive women in Mecha Woreda, North Western Ethiopia. The overall prevalence of alcohol use during pregnancy among reproductive women in the study area was high, with 19 percent of currently pregnant women using it. Previous studies have pinpointed that people in Ethiopia, including pregnant women, have highly consumed both home-made and manufactured alcoholic beverages due to a lack of awareness about the harmful effects of alcohol use and cultural acceptance of alcohol consumption in Kolfe sub-city, Addis Ababa, Ethiopia. As the findings revealed, the prevalence of alcohol consumption, binge-drinking and weekly alcohol consumption of four or more units among pregnant women was 39.78 percent, 3.54 percent and 4.9 percent, respectively [13]. The possible reasons for these discrepancies are variations in the educational status, culture, and socio-economic status of the study population. Additionally, it might be the sample size taken, study setting, or area difference.

According to the results of the logistic regression model, women aged 35 to 49 years decreased their use of alcohol during pregnancy compared to those aged 15 to 24 and 25 to 34 years, respectively. Those women in the age group of 35 to 49 years were less likely to utilize alcohol during pregnancy than those in the age group of 15 to 24 years old, and there was a negative relationship with the utilization of alcohol during pregnancy. Hence, a woman’s age is a significant and negative relationship towards utilizing or consuming alcohol during pregnancy (AOR = 0.221; 95% CI = 0.057–0.856). Alcohol consumption is known to be higher in younger women. Our finding is similar to studies conducted in Geneva [14] and Southeastern Nigeria [15]. This might be because age-related drinking of alcohol may decrease in times of pregnancy.

Illiterate women have a more positive relationship with the utilization of alcohol during pregnancy as compared to women who are literate. These study findings showed that illiterate women were 2.697 times more likely to use alcohol during pregnancy than those literate (AOR = 2.697; 95% CI = 1.207–6.026). This study’s findings are in line with the previous study done in Ethiopia [16]. The rationale for this significant association might be that educated women have good awareness about the risk of drinking alcohol during pregnancy, and they might have experienced behavioral change through learning. The study also demonstrated that women who are pregnant currently have lower alcohol utilization at the time of pregnancy as compared to non-pregnant women. This means that women who are currently pregnant were less likely to use alcohol than non-pregnant women (AOR = 0.139; 95% CI = 0.057–0.343). As a result, pregnancy status was found to be negatively associated with alcohol consumption during pregnancy. Alcohol consumption among reproductive women is a public health concern, considering its adverse outcomes for both the mother and the developing fetus. The rates of alcohol use during pregnancy in the US remain surprisingly high [17, 18]. Generally, South Africa has one of the highest levels of alcohol consumption due to the activities of hazardous and harmful drinking [19]. The study done in Ghana indicated that there is a higher prevalence of current use of alcohol at 49.7 percent [20]. Our study findings are in line with the study done on alcohol consumption during or outside pregnancy as a modifiable risk factor [21]. This might be permanent because women who have a high adherence to alcohol consumption during pregnancy risk putting their lives and fetuses at risk.

The study revealed that women who currently use alcohol have a decreased utilization of alcohol as compared to those who have ever used alcohol (AOR=0.021; 95% CI=0.009-0.049). Hence, women who currently use alcohol had a negative relationship with alcohol use during pregnancy in the study area. But other study findings revealed that women currently using alcohol have a significant and positive relationship with alcohol utilization during pregnancy. The finding of the study is parallel to that done in the Dodoma region, Tanzania, where having ever consumed an alcoholic herbal brew was associated with consumption of alcoholic drinks in the three months preceding the survey [22].

The study findings indicated that the use of alcohol in pre-pregnancy is less likely to utilize alcohol. This means that those reproductive women who have experience of alcohol use in pre- pregnancy have a lower probability of using it than women who have no experience of alcohol use in pre-pregnancy (AOR=0.016; 95% CI=0.006-0.042). The study findings showed that the use of alcohol in pre- pregnancy is statistically significant for reproductive women to utilize alcohol. Our study findings are consistent with the previous studies done in South Africa, northern Uganda, and South-Eastern Nigeria where pre-pregnancy alcohol use is a factor and significantly associated with alcohol use [23–25].

The study also revealed that women who drank alcohol with their husbands during pregnancy were less likely to use alcohol. This means that those women who drink alcohol with their husbands have decreased their use of alcohol as compared to women who do not drink with their husbands (AOR=0.228; 95% CI=0.085-0.614).A previous study conducted in South Africa found that drinking alcohol during pregnancy is a factor that is significantly associated with alcohol use [23].

These study results showed that women who perceive the risk of alcohol consumption during pregnancy as low can utilize alcohol less than women who perceive the risk of alcohol consumption as high (AOR=0.262;95% CI=0.074-0.925). The study also showed that the attitudes of women towards alcohol consumption during pregnancy are medium and can have a higher probability of alcohol consumption as compared to a high perception of alcohol consumption (AOR=0.296; 95% CI=0.103-0.849). A previous study affirmed that the risk of alcohol consumption was associated with the consumption of alcoholic drinks in the three months preceding the survey [22].

The study showed that the attitude of women toward alcohol consumption during pregnancy was a significant factor in this study. Attitudes were strongly associated with alcohol use during pregnancy [25]. The strongest predictors for holding harmful attitudes were a history of abuse and drinking during pregnancy [24]. Most of the members of the community are concerned about women drinking alcohol during pregnancy. To minimize the level of alcohol misuse and encourage people to seek help at an early stage, a societal shift in attitudes toward drinking is required [26].Finally, the study found that women who think that drinking alcohol during pregnancy is beneficial are less likely to drink alcohol than women who disagree, with an odds ratio of (AOR= 0.104; 95 percent CI=0.013-0.833).At a p value of 0.05, this means that the level of agreement or attitude of women is statistically significant and that there is a negative association. Alcohol consumption has been linked to the perception of alcohol as good or desirable [22]. Respondents who had negative attitudes toward alcohol (those who stated it was not all that significant and those who believed it was slightly important in increasing the odds of health concerns for women and babies) were more likely to drink during pregnancy [25].

### Limitation of the study

This study might have limitations in target groups, area coverage, and sample size. Firstly, even though both pregnant and non-pregnant women participated, the interviewed women who were asked about their past pregnancy might have had a recall bias. The study also covered both rural and urban areas, but it was not conducted on a comparative basis, rather than to understand the prevalence and determinants of alcohol use during pregnancy. Additionally, the researchers believed that the sample size was too small, which would be better if the sample were above 800 participants to represent the total population of the study. Next time, researchers will address all the issues raised.

## Conclusion

The results showed that the prevalence of alcohol consumption among reproductive women was high during pregnancy in the study area. Likewise, the women’s age category of 35 to 49 years old, the educational status of illiteracy, currently pregnant women, currently using alcohol, alcohol consumption in times of pre-pregnancy, drinking alcohol with their husband during pregnancy, attitude towards the risk of alcohol consumption during pregnancy at the level of low and medium, and those women who agree that utilization of alcohol during pregnancy is valuable were the factors that determined reproductive women’s alcohol consumption during pregnancy in the study area. There are many factors that do not significantly determine women’s use of alcohol; this might be due to different circumstances and contexts.

### Recommendations

- Based on the findings of this study, the following points are forwarded:
- Healthcare and social affairs professionals should provide awareness of transference towards alcohol utilization effects to change the attitudes of the community in general and women and girls in particular.
- The government should provide home-to-home and health care centers for discussions on maternal health and child health care, especially during pregnancy and its nurture.
- Regional government and social affairs practitioners should transform the harmful and cultural taboos and customs that are inculcated as useful, including alcohol utilization during pregnancy and the habit of substance abuse, that influence the reproductive health of women in both rural and urban areas.

## Data Availability

The current study is not publicly available due to ethical constraints and personal data protection, but the data is available from the corresponding author on reasonable request.

## Abbreviations

AOR: Adjusted odds ratio
CI: Confidence interval
COR: Crude odds ratio
SPSS: Statistical package of social sciences
SD: Standard deviation
USD: United States dollar
